# All-polyethylene versus metal-backed posterior stabilized total knee arthroplasty: similar 2-year results of a randomized radiostereometric analysis study

**DOI:** 10.1080/17453674.2019.1668602

**Published:** 2019-09-25

**Authors:** Shaho Hasan, Perla J Marang-Van De Mheen, Bart L Kaptein, Rob G H H Nelissen, Sören Toksvig-Larsen

**Affiliations:** a Department of Orthopaedics, Leiden University Medical Center, Leiden, The Netherlands;; b Department of Biomedical Data Sciences, Leiden University Medical Center, Leiden, The Netherlands;; c Department of Orthopaedics, Hässleholm Hospital, Hässleholm, Sweden and Department of Clinical Sciences, Lund University, Lund, Sweden

## Abstract

Background and purpose — The all-polyethylene tibial (APT) component, introduced in the early 1970s, was surpassed by metal-backed tibial (MBT) trays as the first choice for total knee arthroplasty (TKA). With improved polyethylene, the modern APT components can reduce costs, and have shown equivalent results in survivorship and early migration of the cruciate-retaining and cruciate-stabilizing designs. This study compares the 2-year migration of a similarly designed APT-posterior stabilized (PS) and a MBT-PS TKA, using radiostereometric analysis (RSA).

Patients and methods — 60 patients were randomized to receive either an APT Triathlon PS or an MBT Triathlon PS TKA (Stryker, NJ, USA). Migration measured by RSA and clinical scores were evaluated at baseline and at 3, 12, and 24 months postoperatively. Repeated measurements were analyzed with a linear mixed model and generalized estimating equations.

Results — The mean maximum total point movement (MTPM) at 3, 12, and 24 months was 0.41 mm (95% CI 0.33–0.50), 0.57 mm (0.44–0.70), and 0.56 mm (0.42–0.69) respectively in the MBT group and 0.46 mm (0.36–0.57), 0.61 mm (0.49–0.73), and 0.64 mm (0.50–0.77) in the APT group. 2 MBT and 1 APT implant were considered unstable at the 2-year follow-up. The KSS Knee score and KSS Function across 3, 12, and 24 months were comparable in both groups.

Interpretation — For an APT-PS designed component, MTPM measured with RSA is comparable to the MBT-PS component after 2 years of follow-up. No differences in complications or clinical outcomes were found.

Despite the many advantages of the all-polyethylene tibial (APT) component, such as avoiding backside wear, preserving tibial bone, and lower costs, it accounts for only 0.1–13% of the total knee arthroplasties (TKA) registered (Norwegian Arthroplasty Register 2018, Swedish Knee Arthroplasty Register 2018). When TKA was introduced in the early 1970s, implants included APT components, but this design was soon replaced by a metal-backed tibial (MBT) component due to disappointing survival rates of the APT (Steinberg and Steinberg 2000, Browne et al. 2011). However, the APT is now regaining interest due to the higher costs of the MBT (Gioe and Maheshwari 2010, Chambers et al. 2016). Furthermore, APT has comparable results to MBT (Gioe and Maheshwari 2010). The advantage of the APT is that it preserves tibial bone as less resection is needed for the same polyethylene thickness, and that it avoids backside wear (Gioe and Maheshwari 2010, Browne et al. 2011, Cheng et al. 2012, Gustke and Gelbke 2017).

Several studies have compared the outcomes of more recent APT designs with MBT in terms of survival, revision, and complications. Although reporting differing results, most studies found comparable survival rates of the APT and MBT (Browne et al. 2011, Cheng et al. 2011, Nouta et al. 2012b, Voss et al. 2016, Longo et al. 2017). Radiostereometric analysis (RSA) objectively measures migration of a prosthesis and can predict revision for aseptic loosening after 2 years (Ryd et al. 1995, Pijls et al. 2012). Few RSA studies comparing the APT and MBT have been conducted, showing less migration for the APT design in 1 study (Van Hamersveld et al. 2018), whilst others found no difference (Adalberth et al. 2000, 2001, Norgren et al. 2004, Hyldahl et al. 2005), but these studies only included cruciate-retaining (CR) or condylar-stabilizing (CS) TKA and not posterior-stabilizing (PS) TKAs. The use of PS designed TKAs varies and is particularly popular in the United States and the Netherlands where it comprises 49% and 56% of all TKAs used, respectively (American Academy of Orthopedic Surgeons 2018, Dutch Arthroplasty Register 2018). The cam-post design of a PS insert could cause additional stress on the tibial component compared with a CR design (Garling et al. 2005, Molt and Toksvig-Larsen 2014). So apart from mixed results in studies with CR and CS designs, outcomes of these studies cannot be extrapolated to PS implants because of this cam-post design. A study comparing PS designed APT and MBT components is therefore needed.

Hence we compared the migration of an APT- versus a MBT-PS designed prosthesis with up to 2-year follow-up using RSA.  

## Patients and methods

This study was a randomized RSA trial comparing the APT-PS Triathlon Total Knee System with the MBT-PS Triathlon (Stryker, Warsaw, USA). Between November 2014 and June 2015, 60 consecutive patients were included and randomized to either an APT-PS or a MBT-PS component at the Hässleholm Hospital (Sweden). A blocked, computer-generated randomization scheme with a 1:1 ratio was used for randomization with a block size of 20. Patients were blinded to the treatment allocation and remained blinded throughout the study. Surgery was performed by 2 orthopedic surgeons who opened sealed opaque envelopes on the day of surgery. Clinical scores were assessed by blinded physical therapists. Inclusion criteria were patients with a painful knee resulting from osteoarthritis who were scheduled to undergo primary total knee surgery and were willing to sign an informed patient consent form. Main exclusion criteria were BMI > 40, a flexion or varus/valgus contracture > 15°, preoperative knee score > 70, and patients who could not make the follow-up visits because of living far away from the hospital.

### Prosthesis and surgical procedure

The Triathlon APT is made from conventional polyethylene, sterilized with gamma radiation in vacuum and is packaged in nitrogen gas (N2Vac). The modular MBT component uses a highly cross-linked polyethylene insert (X3, Stryker Orthopaedics, Mahwah, NJ, USA). Patients were operated in concordance with the surgical protocol using a midline incision and a medial parapatellar approach. No tourniquet was used. Smartset GHV bone cement (DePuy CMW, Blackpool, UK) was applied only to the tibial baseplate. Perioperatively, 8 well-scattered tantalum beads (ø 0.8 mm; RSA Biomedical, Umeå, Sweden) were inserted into the tibial bone as reference markers. 5 beads were inserted into the polyethylene insert of the MBT and in a similar position in the polyethylene of the APT. Patellae were reshaped. The postoperative regime included immediate full weight-bearing and there were no differences in postoperative treatment between the 2 groups.

### Outcome measures

Primary outcome measure was prosthetic migration after 2 years measured by RSA defined as the maximum total point movement (MTPM), which is the length of the translational vector of the marker with the greatest migration in translation or rotation along the transverse, longitudinal, or sagittal axis. In concordance with the ISO 16087 Standard (ISO16087:2013(E), 2013), migration of a left-sided patient will be transformed to match the data of a right-sided patient to enable comparison between patients. Translations and rotations are expressed according to the right-hand screw rule. RSA radiographs were taken with the patient in supine position and the knee in a calibration cage using a biplanar technique at a 90-degree angle (Cage 10, RSA Biomedical, Umeå, Sweden). Radiographs were taken within 1–2 days postoperatively and at 3, 12, and 24 months. The first postoperative examination was taken as reference for subsequent examinations. At 12 months, double measurements were made to determine the precision of the examination. As no migration is expected between these 2 examinations performed at the same point in time, any migration measured will be the measurement error. The precision is expressed as the standard deviation of these measurements. Marker-based analysis using the software model-based RSA version 4.11 (RSAcore, Leiden, the Netherlands) was used. A mean error of rigid body fitting below 0.35 mm and a condition number below 120 were set as cut-off points. A marker configuration model was used if not enough markers were visible at any follow-up moment (Kaptein et al. 2005). Individual prostheses were considered stable if the increase in MTPM between 1 and 2 years postoperatively was ≤ 0.2 mm, and consequently any prosthesis with an MTPM increase of > 0.2 mm was considered as at risk for loosening (Ryd et al. 1995).

Secondary outcome measures were the Knee Society Score (KSS), the Knee Osteoarthritis Outcome Score (KOOS) and the Forgotten Joint Score (FJS). The KSS and KOOS were measured preoperatively and at 3, 12, and 24 months. The FJS was measured at 3, 12, and 24 months. All scores ranged from 0 to 100 with higher scores indicating better scores.

### Sample size

Sample size was calculated assuming that a difference of 0.3 mm for translation and 0.25° for rotation would be clinically relevant. 17 patients were needed in each group with an alpha of 0.05 and a power of 0.80. Taking into account that patients with inappropriate marking of the prosthesis or tibial bone will be excluded as well as possible patients lost to follow-up, 30 patients were included in each group.

### Statistics

Analyses were performed according to the intention-to-treat principle. MTPM, translations, rotations, and clinical outcome scores were analyzed with a linear mixed model if normally distributed. This model is recommended to analyze repeated measurements as it takes the within-subject correlation as well as the missing values into account (Ranstam et al. 2012). The model consisted of a group variable (APT versus MBT), a time variable (baseline, 3 months, 12 months, and 24 months), and an interaction term (fixed effects). An Autoregressive Order-1 covariance matrix was used to model remaining variability. The generalised estimating equations (GEE) approach was used if a normal distribution could not be obtained through transformation. This approach was needed for the analysis of MTPM, the KSS Knee score and the KOOS Sports subscore. Mean translations and rotations are reported per group at 3, 12, and 24 months. Mean scores of the KSS Knee, KSS Function, and the 5 subscales of the KOOS are reported per group preoperatively, and at 3, 12, and 24 months postoperatively. The mean FJS is reported at 3, 12, and 24 months postoperatively. P-values < 0.05 were considered statistically significant. Means are reported with 95% confidence intervals (CI). Analyses were performed with SPSS version 23 (IBM SPSS Statistics 23.0; IBM Corp, Armonk, NY, USA).

### Ethics, registration, funding, and potential conflicts of interest

Approval of the Regional Ethical Review Board in Lund was obtained before recruitment (entry no. 2014/513). This study was registered at the ISRCTN Registry (ISRCTN10744502) and was conducted in accordance with the CONSORT statement. All patients provided informed consent. Stryker funded this study but did not take any part in the design, conduct, analysis, and interpretations stated in this paper.  

## Results

**Figure 1.**
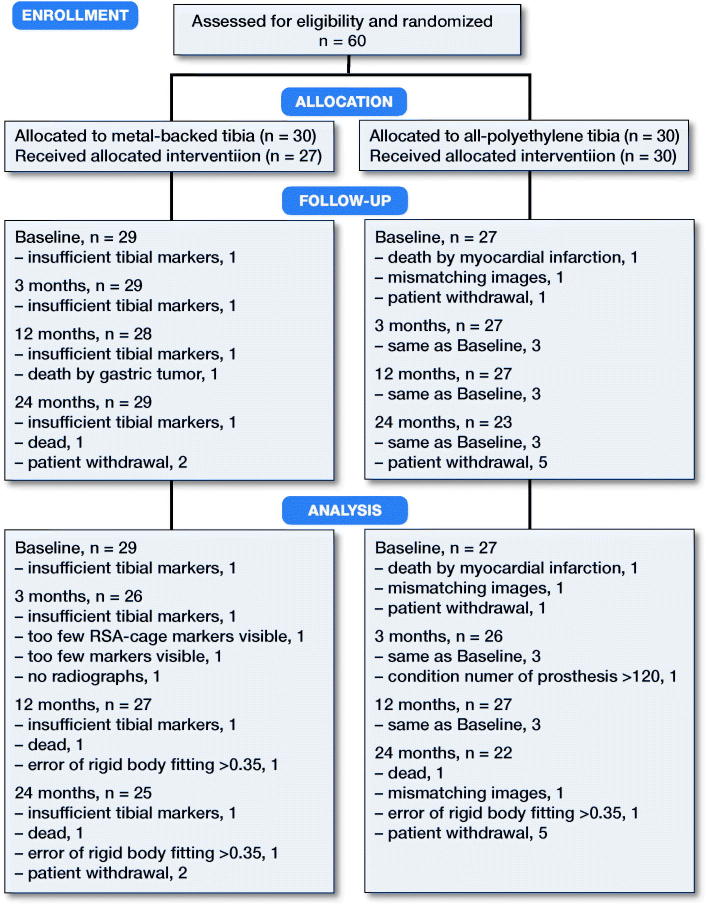
CONSORT flow chart.

60 patients were included and randomized to either the APT-PS or the MBT-PS total knee prosthesis. After randomization, 4 patients were excluded. 56 patients were thus included in the analysis (Figure 1). During follow-up, 9 patients withdrew or had radiographs that could not be analyzed, leaving 47 patients for analysis at 2 years (Figure 1). Age, BMI, sex, ASA score, and Ahlbäck classification were similar at baseline. Each surgeon operated on approximately half of the patients in both groups. Postoperatively, the MBT implants seemed to be more in varus compared with the APT (Table 1).

**Table 1.** Baseline demographic characteristics. Values are frequency unless otherwise stated

Factor	Metal-backed	All-polyethylene	Total
Patients	29	27	56
Age, mean years (SD)	68 (4)	68 (4)	68 (4)
BMI, mean (SD)	28 (4)	29 (3)	28 (3)
Sex			
Female	17	13	30
Male	12	14	26
ASA classification			
I	4	7	11
II	18	17	35
III	7	3	10
Surgeon			
#1	14	14	28
#2	15	13	28
Ahlbдck classification			
II	5	4	9
III	23	23	46
IV	1	0	1
HKA postoperative			
Varus (< 177°)	7	3	10
Neutral (177–183°)	15	17	32
Valgus (> 183°)	2	4	6
Missing **^a^**	5	3	8

SD = standard deviation, HKA = hip–knee–ankle angle.

a Some patients had no postoperative long-leg radiographs taken and HKA could not be assessed.

**Figure 2.**
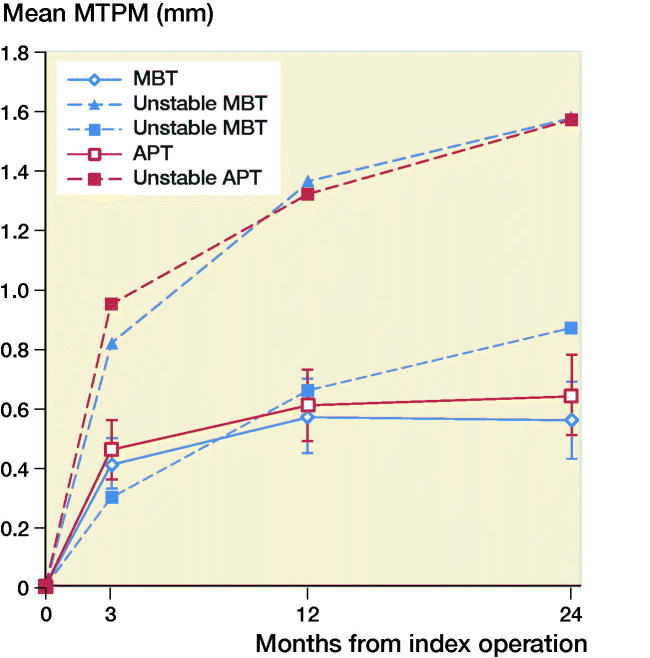
Mean (95% CI) MTPM in mm of the metal-backed tibial implant group (MBT) and the all-polyethylene tibial implant group (APT) at 3, 12, and 24 months follow-up. The MTPM of the 3 unstable implants is plotted and all 3 show continuous migration between 12 and 24 months’ follow-up.

**Figure 3.**
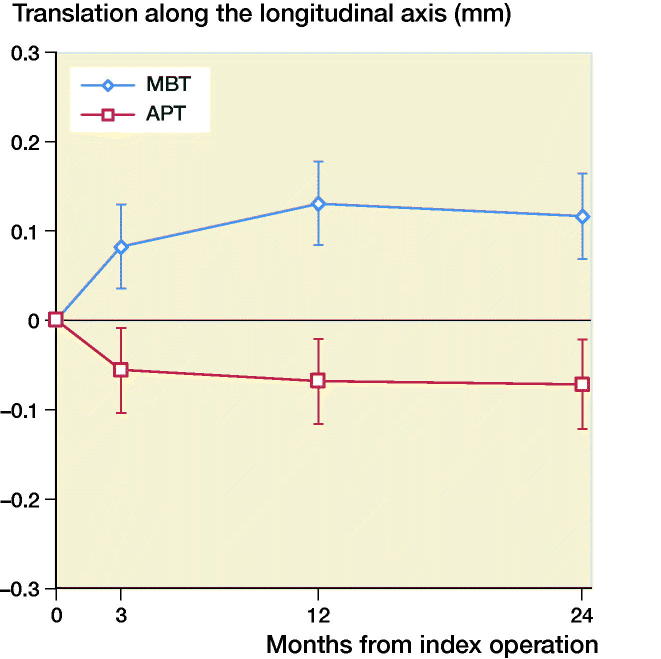
Mean translation along the longitudinal axis in mm with 95% confidence intervals. A positive value indicates tibial lift-off and a negative value indicates subsidence of the tibial implant.

**Figure 4.**
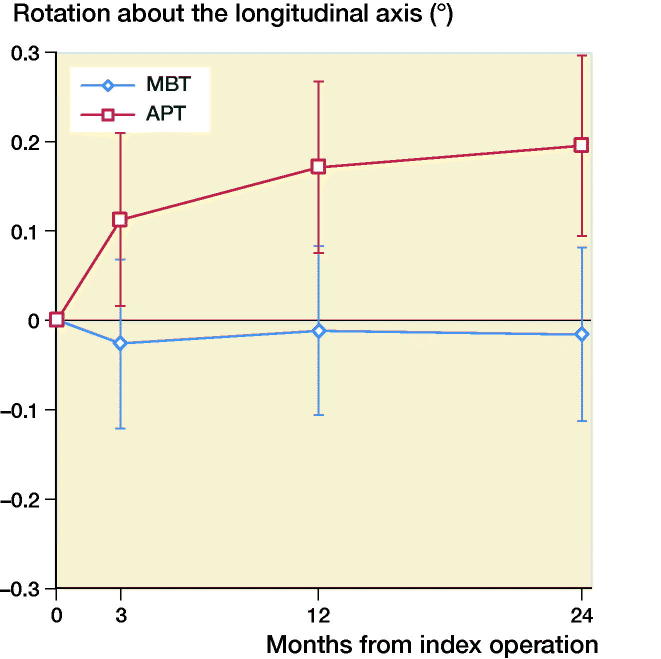
Mean rotation along the longitudinal axis in degrees with 95% confidence intervals. A positive value indicates internal rotation and a negative value indicates external rotation of the tibial implant.

**Figure 5.**
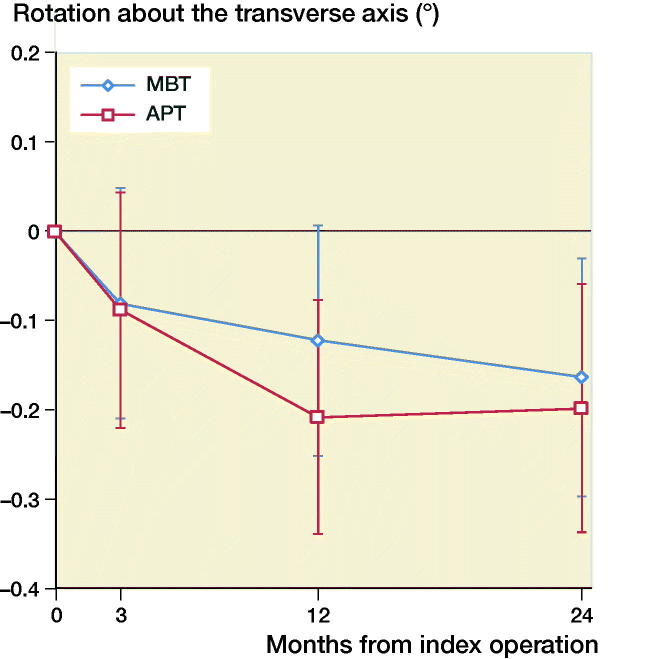
Mean rotation along the transverse axis in degrees with 95% confidence intervals. A positive value indicates forward tilting and a negative value indicates backward tilting of the tibial implant.

The mean MTPM across 3, 12, and 24 months was similar in both groups. The mean MTPM change from 12 to 24 months was –0.01 mm (CI –0.19 to 0.17) in the MBT group and 0.03 (CI –0.14 to 0.21) in APT group (Table 2; Figure 2). 2 implants in the MBT and 1 in the APT group displayed > 0.2 mm MTPM between 1- and 2-year follow-up, and were considered unstable (Figure 2). The MBT group showed lift-off (positive), while the APT group showed tibial subsidence (negative) (Figure 3, Translation along the longitudinal axis). A different migration pattern between the groups was also visible in the rotation along the longitudinal axis, being external (negative) in the MBT and internal (positive) in the APT group (Figure 4, Rotation about the longitudinal axis). Other translations and rotations were similar between groups with backward tilting (negative) being the most prominent direction of migration in both groups (Figure 5, Rotation along the transverse axis; Figures 6–8, see Supplementary data). None of the patients were scheduled for revision surgery. 50 double measurements were made at 1-year follow-up. The precision of the measurements of the translations and rotations were 0.1 mm and 0.1 degrees. The mean condition number of the tibial bone and the prosthesis was 42 (range 20–108) and 40 (range (21–114), respectively. The mean error of rigid body fitting was 0.14 (range 0.04–0.34) and 0.08 (range 0.01–0.35) of the tibial bone and the prosthesis, respectively.

**Table 2.** Mean (95% CI) MTPM in mm of the metal-backed tibial implant group (MBT) and the all-polyethylene tibial implant group (APT) at 3, 12, and 24 months follow-up

Time (months)	MBT	APT
3	0.41 (0.33–0.50)	0.46 (0.36–0.57)
12	0.57 (0.44–0.70)	0.61 (0.49–0.73)
24	0.56 (0.42–0.69)	0.64 (0.50–0.77)

The KSS Knee scores across 3, 12, and 24 months were similar in both groups. KSS Function score was also similar. Moreover, no statistically significant difference was found in the KOOS subscores or in the FJS (Table 3, see Supplementary data).

## Discussion

We found similar MTPM between the APT-PS and MBT-PS at 2-year follow-up; the translation and rotation along and about the 3 orthogonal axes were different for longitudinal translation and rotation. Van Hamersveld et al. (2018), who used a CS design, and other RSA studies on CR designs reported comparable MTPM values to those in our study (Adalberth et al. 2000, 2001; Norgren et al. 2004; Hyldahl et al. 2005). These findings suggest that, although PS implants most likely experience different shear forces at the implant–bone interface, the MTPM values after 2-year follow-up are comparable to CR and CS designs. Furthermore, despite the relative elasticity of a full APT component, this did not result in a difference in migration compared to a MBT component. This may imply that the polyethylene insert within the metal baseplate gives enough peak stress absorption in the PS design. The difference in translation along the longitudinal axis was previously described by Adalberth et al. (2000) who compared a low-conforming APT and MBT with RSA, and concluded that this finding might be explained by an increase in tensile forces in the less flexible MBT (Bartel et al. 1982). In our study, the subsidence of the APT and lift-off of the MBT stabilized after 3 months. The difference in rotation about the longitudinal axis (i.e., internal/external rotation) between the MBT and APT in our study might be due to unmeasured differences between the groups such as the alignment of the tibial component. Another explanation might be the minor differences in the postoperative HKA between groups, but the groups are too small to draw any valid conclusion. We reported signed migration values in contrast to several other RSA studies. In order to allow comparison between RSA studies and to understand the direction of migration, reporting signed values is preferred as was previously suggested by Valstar et al. (2005).

Gudnason et al. (2017) suggested that it was better to use the transversal rotation for analysis of RSA migration data as it was a better predictor for aseptic loosening than MTPM. The rotation in the transverse plane was posterior for both groups (Figure 5, Rotation along the transverse axis). The posterior rotation of the tibial implants in both groups could be due to anterior engagement of the cam-post mechanism of the PS design, which engages in extension. Banks et al. (2002) found that TKAs are frequently aligned in relative hyperextension, which might explain the rotation in the present study. Another factor contributing to the posterior rotation might be the single-radius design of the TKA used in our study, which might play a role as the center of rotation lies more posteriorly compared with multi-radius designs (D’Lima et al. 2001). Whether this migration pattern has clinical consequences remains unclear and should be studied further when longer follow-up data become available.

The KSS Knee and Function scores increased postoperatively and were comparable in both groups during follow-up, which is consistent with previous studies (Adalberth et al. 2000, 2001). The KOOS subscales and the FJS also showed similar results. De Carvalho et al. (2013) used different clinical outcomes (the Oxford Knee Score, the Western Ontario and McMaster Universities Arthritis Index, and the Short form-12 scores), but also found no difference between groups.

Our study with an all-polyethylene PS design failed to show superiority of either APT or MBT. Nevertheless, Chambers et al. (2016) estimated that a reduction of 42% in costs could be achieved if the APT were used. However, the actual costs of an implant differ widely and the total costs of TKA treatment consist of more than just the tibial component, including personnel, equipment, and space costs. In addition, the financial benefit of the APT might not outweigh the limitations as it cannot be coated and liner exchange is not possible. These factors may be among the reasons why orthopedic surgeons continue to use the MBT TKA as the implant of first choice even though some suggest that the APT could be an acceptable treatment in patients above 70 years of age or with rheumatoid arthritis (Nouta et al. 2012a).

A limitation of this study is the lack of power to detect a difference in clinical scores between the two groups. RSA studies, in general, include small groups and probably fail to detect any differences due to this small sample size. Including more patients, however, would nullify the strength of RSA studies as it can measure migration with high precision and, therefore, only a small sample size is needed to assess the stability of implants. Another limitation is the difference in polyethylene as the polyethylene insert of the MBT tray was made of highly crosslinked polyethylene and the APT was made from conventional polyethylene. Ideally, the polyethylene in both implants would be the same, but this was not possible due to manufacturing limitations.

In summary, the APT-PS TKA prosthesis has comparable migration to the MBT-PS TKA in terms of MTPM measured by RSA at 2 years of follow-up, even though there was a different pattern in longitudinal translation and rotation. No differences in complications or clinical outcomes were found between the two groups. 

### Supplementary data

Table 3 and Figures 6–8 are available as supplementary data in the online version of this article, http://dx.doi.org/10.1080/ 17453674.2019.1668602

## Supplementary Material

Supplemental Material
