# Supplementary material for: All-polyethylene versus metal-backed posterior stabilized total knee arthroplasty: similar 2-year results of a randomized radiostereometric analysis study
Source: Acta Orthop. 2019 Sep 25;90(6):590–5. doi: 10.1080/17453674.2019.1668602 (PMC6844393; doi:10.1080/17453674.2019.1668602)
Supplement: Supplemental Material [file IORT_A_1668602_SM8914.pdf]

Supplementary data

Table 3. Mean clinical outcome scores with 95% confidence intervals

Factor	MBT	APT
KSS Knee		
Preoperative	46 (43–50)	44 (42–46)
3 months	93 (91–95)	86 (81–91)
12 months	93 (90–96)	94 (90–97)
24 months	98 (96–100)	95 (90–99)
KSS Function		
Preoperative	54 (49–59)	52 (47–58)
3 months	73 (68–78)	76 (71–82)
12 months	85 (80–90)	85 (80–90)
24 months	88 (83–93)	82 (77–88)
KOOS Symptoms		
Preoperative	47 (41–53)	49 (42–55)
3 months	67 (61–73)	62 (56–68)
12 months	77 (71–83)	72 (66–79)
24 months	80 (73–86)	75 (68–82)
KOOS Pain		
Preoperative	38 (31–45)	41 (34–48)
3 months	70 (63–77)	66 (59–73)
12 months	82 (75–88)	80 (73–87)
24 months	87 (80–94)	79 (72–86)
KOOS ADL		
Preoperative	42 (35–48)	45 (38–52)
3 months	75 (68–81)	67 (61–74)
12 months	80 (73–86)	75 (68–82)
24 months	82 (75–89)	75 (68– 82)
KOOS Sports		
Preoperative	9 (5–13)	12 (8–17)
3 months	35 (26–44)	21 (14–29)
12 months	42 (33–52)	40 (30–50)
24 months	42 (35–50)	40 (29–50)
KOOS QoL		
Preoperative	33 (27–38)	35 (30–40)
3 months	46 (41–51)	43 (38–48)
12 months	55 (50–60)	53 (48–58)
24 months	54 (49–60)	53 (48–59)
FJS		
3 months	36 (25–46)	35 (24–46)
12 months	61 (50–72)	56 (45–67)
24 months	58 (47–69)	51 (40–63)

KSS = Knee Society Score, KOOS = Knee Osteoarthritis Outcome Score, ADL = activities of daily living, QoL = quality of life, FJS = forgotten joint score.

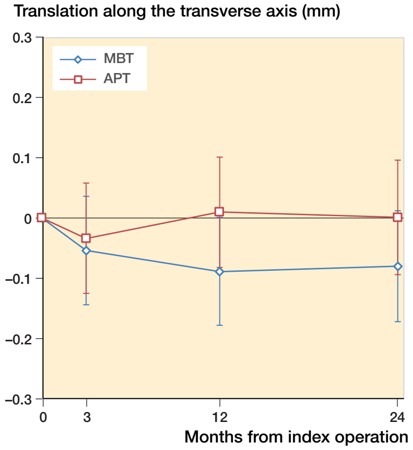

Figure 6. Mean translation along the transversal axis in mm with 95% confidence intervals. A positive value indicates medial translation and a negative value indicates lateral translation of the tibial implant.

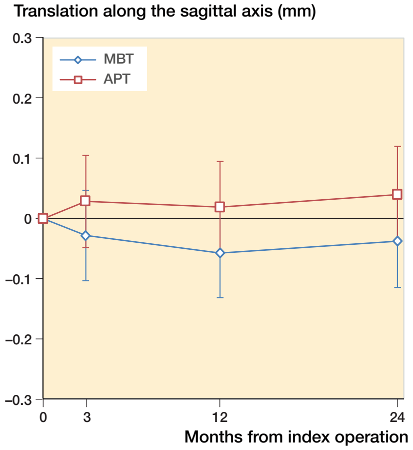

Figure 7. Mean translation along the sagittal axis in mm with 95% confidence intervals. A positive value indicates anterior translation and a negative value indicates posterior translation of the tibial implant.

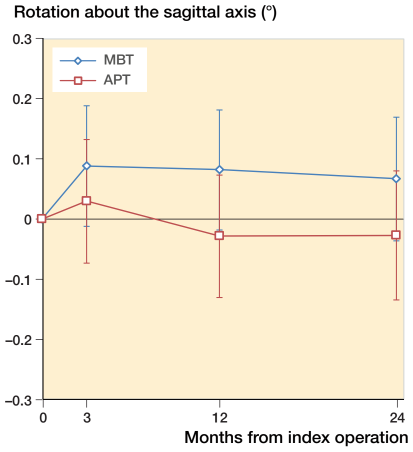

Figure 8. Mean rotation along the sagittal axis in degrees with 95% confidence intervals. A positive value indicates medial tilting and a negative value indicates lateral tilting of the tibial implant.
